# The impact of anxiety and depression on the quality of life of hemodialysis patients in a sample from Somalia

**DOI:** 10.1186/s12888-023-05312-8

**Published:** 2023-11-10

**Authors:** Nur Adam Mohamed, Asir Eraslan, Samet Kose

**Affiliations:** 1 Department of Psychiatry and Behavioral Sciences, Mogadishu Somalia Türkiye Recep Tayyip Erdogan Research and Training Hospital, Mogadishu, Somalia; 2 Department of Urology, Mogadishu Somalia Türkiye Recep Tayyip Erdogan Research and Training Hospital, Mogadishu, Somalia

**Keywords:** End-stage kidney disease, Hemodialysis, Anxiety, Depression, Quality of life, Somalia

## Abstract

**Objective:**

The main objective of the present study was to determine the quality of life (QoL), depression, and anxiety levels in kidney failure patients undergoing hemodialysis (HD) treatment and examine the impact of depression and anxiety on the QoL of these patients in a sample from Somalia.

**Methods:**

A sample of 200 patients with kidney failure who were undergoing HD treatment approximately two to three times a week was included. All participants were administered a sociodemographic data form, the Patient Health Questionnaire-9 (PHQ-9), the Hospital Anxiety and Depression Scale (HADS), and the World Health Organization Quality of Life (WHO-QOL-BREF). Subjects on HD for less than 3 months prior to the study date were excluded.

**Results:**

Of the kidney failure patients, 200 patients, aged 18–86 years (median: 50.0; IQR: 25.0), consented and participated in the study. 58.5% of the participants were men; 64% had a kidney failure duration of 1–5 years, and 52.6% had a HD duration of 1–5 years. Mild symptoms of depression were found in 48%, while moderate-to-severe depressive symptoms were found in 13.5% of HD patients. Depression and anxiety were found to be significantly correlated with overall QoL, physical health, psychological health, social relationships, and environmental well-being. There were similar predictors of overall QoL, physical health, psychological well-being, social relationships, and environmental well-being relating to socio-demographic factors such as age, gender, family income, anxiety, and depression.

**Conclusions:**

This study emphasizes the important role that anxiety, depression, and family income have in HD patients' QoL in Somalia. It highlights the significance of regular emotional assessment and efficient management in order to increase patient satisfaction. Future studies with larger samples are necessary for more accurate statistical analysis. To optimize patient care, a multidisciplinary healthcare team is recommended.

## Introduction

Kidney failure presents a significant health concern because of its severe health impacts, high death and illness rates, and constant need for demanding and frequent medical attention, which can be overwhelming for patients and their families [1]. In many countries lacking public funding for kidney failure care, patients are unable to access standard HD treatment, leading to poor health outcomes and death [2]. Despite advancements in technology and HD accessibility, patients' health decline and poor QoL have remained unchanged from a public health perspective [1]. Poor QoL in HD patients is due to factors such as reduced physical health, emotional well-being, loss of work capacity, financial dependence, the inability to meet family obligations, and limited social involvement [3, 4]. These factors may result in psychiatric disorders such as depression and anxiety as well as decreased cognitive function [3].

In terms of psychiatric disorders, major depressive disorder was reported to be one of the most prevalent forms in kidney failure patients undergoing HD [5, 6]. A study in Nigeria found a 34.5% depression prevalence among HD patients, compared to 27% in African Americans in the US [7] and 45% in a sample from Ghana [8]. The highest depression prevalence among HD patients was reported at 72% in a sample from Sudan [9]. Risk factors for depression in HD patients include poor treatment adherence, inadequate nutrition, low perceived social support, and altered immune system functioning [10]. Longer duration of HD has been linked to higher depression prevalence and poorer QoL [8, 11, 12]. Age and major family problems have also been identified as predictors of anxiety and depression that greatly affect QoL and well-being in HD patients [13].

The prevalence of CKD in Eastern Africa was reported as 14.4%, lower than the average African prevalence of 15.8% (95% CI 12.1–19.9) [14]. However, to the best of our knowledge, the exact number of kidney failure patients receiving HD treatment in Somalia is unknown and under-researched. In this present study, we aimed to determine the QoL and levels of depression and anxiety in kidney failure patients receiving HD treatment and examine the effect of depression and anxiety on QoL in a sample of HD patients.

## Methods

The study design was cross-sectional and was conducted at the HD unit of the Mogadishu Somalia-Türkiye Recep Tayyip Erdogan Training and Research Hospital in Mogadishu, Somalia. The participants were 200 patients (83 women and 117 men) who were undergoing HD treatment approximately two to three times a week. Patients who had been on HD for less than three months prior to the study date were not included. The sample size was calculated using Open Epi version 3.01 by taking alpha 0.05 (95% confidence level), which gave the final sample size of 190 patients [population size (for finite population correction factor or fpc)(*N*): 17,597,511; hypothesized % frequency of outcome factor in the population (*p*): 14.4% ± 5; confidence limits as % of 100 (absolute ± %)(*d*): 5%; design effect (for cluster surveys-*DEFF*): 1]. During data collection, we managed to collect data from 200 patients. All participants were administered a sociodemographic data form, the Patient Health Questionnaire-9 (PHQ-9), the Hospital Anxiety and Depression Scale (HADS), and the World Health Organization Quality of Life (WHO-QOL-BREF). All three assessment tools used in this study are reliable and valid among patients undergoing HD and have been used in previous studies [5, 8, 13]. According to King-Wing Ma and Kam-Tao Li, it is still unclear which screening instrument for depression in dialysis patients is appropriate, and the PHQ-9 is one of the most widely used and validated questionnaires for this purpose [5]. Similarly, as reported by Preljevic et al., HADS has acceptable psychometric properties and can be used as a valid and reliable screening tool for depression and anxiety in dialysis patients [15]. The WHOQoL-BREF questionnaire is also a valid, accurate, and dependable tool for measuring HD patients' QoL [16].

### Psychometric scales

#### Patient Health Questionnaire-9 (PHQ-9)

The PHQ-9 is a 9-item scale developed by Kroenke et al. (2001) [17] that measures the depressive thoughts and feelings of individuals specifically during the previous two weeks. Individuals respond to the items in a range from 0 to 3, 0 for "not at all" and 3 for "nearly every day" responses. Scores range from 0 to 27. A PHQ-9 score < 10 signifies no depression; a score of 10 to 14 signifies a moderate level of depression; and a score ≥ 15 signifies moderate to severe depression. The Cronbach’s alpha of the PHQ-9 of the original study was reported as 0.89 in the primary care patients and 0.86 in the OB/GYN patients. The Somali version of the PHQ-9 [18] reported a Cronbach’s alpha of 0.79. The Cronbach’s alpha for the PHQ-9 in the present sample was 0.76.

#### Hospital Anxiety and Depression Scale (HADS)

The HADS is a 14-item self-report scale developed by Zigmond and Snaith (1983) [19] to determine the presence of anxiety and depression symptoms in non-psychiatric patients receiving treatments at the hospital and outpatient clinics. The HADS consisted of an anxiety dimension (HADS-A) and a depression dimension (HADS-D), with seven items to measure each dimension using a 4-point Likert scale. A score of 0 to 7 is considered normal; 8 to 10 indicates a mild disorder; 11 to 15 indicates a moderate disorder; and a score of 16 to 21 is suggestive of a severe disorder. The Somali version of the HADS [20] reported a Cronbach’s alpha of 0.83 for the anxiety subscale and 0.84 for the depression subscale. The Cronbach’s alpha of the HADS for the present sample was 0.84 for the anxiety subscale and 0.75 for the depression subscale.

#### World Health Organization Quality of Life (WHOQOL-BREF)

The WHOQOL-BREF was developed by the WHO (1998) to determine four major dimensions of QoL: physical health, psychological health, social relationships, and environmental well-being [21]. The WHOQOL-BREF contains 26 items; the first two items report overall QoL and general health. Each dimension uses a 5-point Likert scale from 1 (very poor/very dissatisfied) to 5 (very good/very satisfied). The raw domain scores in the WHOQOL-BREF are transformed and found to be comparable with the scores in the WHOQOL-100. The Somali version of the WHOQOL-BREF [22] reported a Cronbach’s alpha of 0.65 for the physical domain, 0.71 for the psychological domain, 0.76 for the social relationship domain, and 0.82 for the environmental domain. The Cronbach’s alpha of the WHOQOL-BREF for the present sample was 0.65 for the overall WHOQOL-BREF, 0.43 for the physical domain, 0.47 for the psychological domain, 0.47 for the social domain, and 0.70 for the environmental domain. As per the literature, a cutoff point has been established to distinguish between a good QoL (score ≥ 60 cutoff) and a poor QoL (score < 60 cutoff) [23].

### Statistical analysis

All statistical analyses were performed using SPSS (Armonk, NY: IBM Corp.), version 26.0. Analysis and presentation of categorical variables in the form of frequencies and percentages were done. The median and interquartile range were used to display the continuous variables. To determine the normality of the data, preliminary studies were performed. Since the data was not normally distributed, Spearman’s Rank-Order Test was used for correlation analyses. In terms of sociodemographic variables and QoL dimensions, Mann–Whitney’s U test was performed for comparisons between two groups, and the Kruskal–Wallis test was performed for comparisons between more than two groups. The predictive relationship between anxiety and depression levels and QoL parameters was investigated using hierarchical regression analyses, where predictors are entered into the model in a specific order or hierarchy. This means that predictors are added to the model in separate steps, with each step building upon the previous one. In contrast, standard regression models like linear or quantile regression typically involve a single stage of analysis where all predictors are included simultaneously. Hierarchical regression facilitates a more nuanced interpretation of the relationships between variables, as it allows you to evaluate how the inclusion of predictors at each stage changes the model's fit and the coefficients of the predictors. In our model, age, gender, and family income were entered as categorical variables, and duration of kidney failure and duration of HD were entered as continuous variables. Beta coefficients from the hierarchical regression model indicate the median change in the outcome. A p-value of less than 0.05 was accepted as statistically significant.

## Results

The average median age of the 200 participants in the study was 50.0, with an IQR of 25.0. The sample consisted of 41.5% women and 58.5% men undergoing HD treatment. 64% of the participants had a duration of CKD of 1–5 years, and 52.6% had a duration of HD of 1–5 years. In 58.5% of participants, hypertension was reported as the cause of kidney failure, and in 20% of the participants, diabetes mellitus was reported as the cause of kidney failure. The results were displayed in Table 1.

**Table 1:** Sociodemographic characteristics of the study participants (*n* = 200)

Variable	Category	n	%
Age (years)	18–24	9	4.5
	25–34	22	11
	35–44	28	14.0
	45–54	45	22.5
	> 55	96	48.0
Gender	Female	83	41.5
	Male	117	58.5
Marital status	Single	12	6.0
	Married	139	69.5
	Divorced	24	12.0
	Widowed/Widower	25	12.5
Education status	Illiterate	137	68.5
	Intermediate	20	10.0
	Secondary	30	15.0
	University	13	6.5
Occupational status	Unemployed	188	94.0
	Employed	12	6
Family income	Missing	88	44.0
	1000 – 1500 dollars	22	11.0
	1500 – 2000 dollars	45	22.5
	> 2000 dollars	45	22.5
Duration of CKD	< 1 year	40	20.0
	1–3 years	69	34.5
	3–5 years	59	29.5
	> 5 years	32	16.0
Cause of kidney failure	Hypertension	117	58.5
	Diabetes mellitus	40	20.0
	Glomerulonephritis	5	2.5
	Others	38	19.0
Duration on HD	3 months	25	12.5
	1 year	46	23.0
	1–3 years	51	25.5
	3–5 years	55	27.1
	> 5 years	23	11.5
Number of dialysis sessions per week	Once a week	21	10.5
	Twice a week	140	70.0
	Thrice a week	34	17.0
	Four times a week	5	2.5

*CKD* Chronic Kidney Disease, *HD* Hemodiaysis

The median overall scores were 6.0 (IQR: 4.0) for PHQ-9, 2.0 (IQR: 4.0) for HADS-Anxiety, 6.0 (IQR: 6.0) for HADS-Depression, 75.0 (IQR: 25.0) for overall quality of health, 46.4 (IQR: 25.0) for physical health, 70.0 (IQR: 16.7) for psychological health, 25.0 (IQR: 25.0) for social relationships, and 65.6 (IQR: 15.6) for environmental well-being. Among the 200 HD patients, 78.5% scored above 60 on the Overall Quality-General Health scale, while 21.5% scored below 60. For physical health, 31.5% exceeded 60, with 68.5% below. In terms of psychological well-being, a significant 83% scored above 60, while 17% scored below. Social relationships were an area of concern, with only 4.5% achieving scores above 60, while 95.5% fell below this threshold. For environmental well-being, 69.5% scored above 60, while 30.5% scored below. For the PHQ-9 scale, 38.5% of the participants reported no symptoms of depression, 48% reported mild symptoms of depression, and 13.5% of the participants reported moderate-to-severe symptoms of depression. For the HADS-Anxiety scale, 92.5% of the participants reported no symptoms of anxiety, 7% reported mild to moderate symptoms of anxiety, and only 0.5% reported severe symptoms of anxiety. For the HADS-Depression scale, 62.5% of the participants reported no symptoms of depression, and 37.5% reported mild to moderate symptoms of depression.

A Mann–Whitney test indicated that median social relationship scores were significantly greater for men than for women (U = 3524.0, *p* < 0.001). A Kruskal–Wallis test indicated that physical health scores [H(4) = 17.80, *p* < 0.001] and social relationship scores [H(4) = 25.86, *p* < 0.001] were significantly greater for younger patients than for older patients. Married HD patients were found to have significantly higher social relationship scores [H(3) = 12.36, *p* = 0.006] compared to single and divorced patients. Patients with a shorter duration of CKD scored significantly better in physical health [H(3) = 8.20, *p* = 0.042] and psychological well-being [H(3) = 10.28, *p* = 0.016]. Although we expected the duration of HD to have an impact on QoL in HD patients, we did not find any significant results. Patients with a lesser number of dialysis sessions per week scored significantly better in physical health [H(3) = 8.06, *p* = 0.045]. The results were displayed in Table 2.

**Table 2:** Comparison of the WHOQOL-BREF overall and domain scores based on sociodemographic variables (*n* = 200)

**Variable**		**OverallQoL/ General Health**	**Physical**	**Psychological**	**Social Relationships**	**Environmental**
Age (years)	18–24	75.0 (25.0)	60.7 (25.0)	75.0 (14.6)	33.3 (20.8)	68.8 (18.8)
	25–34	75.0 (25.0)	60.7 (18.8)	75.0 (5.2)	41.7 (27.1)	65.6(12.5)
	35–44	75.0 (34.4)	53.6 (26.8)	70.8 (16.7)	41.7 (25.0)	67.2 (14.8)
	45–54	75.0 (18.8)	42.7 (17.9)	70.8 (12.5)	25.0 (20.8)	65.6 (15.6)
	> 55	75.0 (25.0)	46.4 (27.7)	70.8 (12.5)	25.0 (16.7)	65.6(15.6)
	Kruskal–Wallis H	1.66	17.80	6.32	25.86	5.14
	p	0.799	< 0.001	0.176	< 0.001	0.273
Gender	Female	75.0 (25.0)	46.4 (25.0)	70.8 (16.7)	25.0 (16.7)	65.6 (12.5)
	Male	75.0(12.5)	50.0 (26.8)	75.0 (12.5)	33.3 (20.8)	65.6 (15.6)
	Mann–Whitney U	4487.5	4184.5	4471.0	3524.0	4779.0
	p	0.347	0.095	0.337	< 0.001	0.849
Marital status	Single	68.8 (25.0)	58.9 (29.5)	72.9 (28.1)	33.3 (20.8)	68.8 (19.5)
	Married	75.0 (25.0)	46.4 (25.0)	70.8 (12.5)	33.3 (25.0)	65.6 (15.6)
	Divorced	75.0 (25.0)	46.4 (32.1)	72.9 (15.6)	25.0 (25.0)	65.6 (12.5)
	Widowed/Widower	75.0 (18.8)	42.9 (21.4)	70.8 (14.6)	16.7 (20.8)	62.5 (10.9)
	Kruskal–Wallis H	2.08	5.23	4.03	12.36	4.42
	p	0.557	0.155	0.258	0.006	0.220
Education status	Illiterate	75.0 (25.0)	46.4 (25.0)	70.8 (14.6)	25.0(16.7)	65.6 (12.5)
	Elementary	81.3 (12.5)	51.8 (21.4)	75.0 (15.6)	25.0 (25.0)	65.6 (14.8)
	Secondary	75.0 (12.5)	48.2 (28.6)	75.0 (12.5)	33.3 (18.8)	65.6 (19.5)
	University	87.5 (18.8)	71.4 (23.2)	83.3(14.6)	50.0(25.0)	78.1 (15.6)
	Kruskal–Wallis H	16.76	12.02	21.45	17.64	9.97
	p	< 0.001	0.007	< 0.001	< 0.001	0.019
Occupational status	Unemployed	75.0 (25.0)	46.4 (21.4)	70.8 (16.7)	25.0 (25.0)	65.6 (12.5)
	Employed	75.0 (21.9)	71.4 (8.9)	79.2 (16.7)	37.5 (37.5)	73.4 (11.7)
	Kruskal–Wallis H	1.71	22.37	6.59	3.58	6.06
	p	0.425	< 0.001	0.037	0.167	0.048
Family income	1000—1500 dollars	68.8 (25.0)	53.6 (26.8)	68.8 (17.7)	25.0 (29.2)	64.1 (18.8)
	1500—2000 dollars	75.0 (25.0)	50.0 (33.9)	70.8 (14.6)	25.0 (25.0)	62.5 (12.5)
	> 2000 dollars	75.0 (18.8)	46.4 (26.8)	75.0 (12.5)	33.3 (25.0)	75.0 (20.3)
	Kruskal–Wallis H	7.71	1.90	1.80	3.29	11.99
	p	0.021	0.388	0.406	0.193	0.002
Duration of CKD	< 1 year	75.0 (21.9)	53.6 (25.0)	75.0 (11.5)	33.3 (39.6)	65.6 (15.6)
	1‑3 years	75.0 (25.0)	50.0 (32.1)	70.8 (12.5)	25.0 (25.0)	65.6 (10.9)
	3‑5 years	75.0 (25.0)	46.4 (17.9)	70.8 (12.5)	25.0 (16.7)	65.6 (18.8)
	> 5 years	75.0 (25.0)	39.3 (21.4)	68.5 (16.7)	25.0 (16.7)	65.6 (14.1)
	Kruskal–Wallis H	0.84	8.20	10.28	4.07	2.44
	p	0.841	0.042	0.016	0.254	0.487
Duration of HD	3 months	75.0 (25.0)	53.6 (23.2)	75.0 (10.4)	33.3 (29.2)	65.6 (15.6)
	1 year	75.0 (25.0)	53.6 (30.4)	70.8 (17.7)	25.0 (25.0)	65.6 (10.2)
	1‑3 years	75.0 (25.0)	50.0 (25.0)	70.8 (12.5)	25.0 (25.0)	68.8 (15.6)
	3‑5 years	75.0 (25.0)	46.4 (17.9)	70.8 (16.7)	25.0 (16.7)	65.6 (21.9)
	> 5 years	73.4 (12.7)	39.3 (21.4)	70.8 (12.5)	25.0 (16.7)	68.8(12.5)
	Kruskal–Wallis H	2.50	6.10	5.76	5.15	4.88
	p	0.644	0.192	0.218	0.272	0.300
Number of dialysis sessions per week	Once a week	75.0 (12.5)	46.4 (25.0)	70.8 (16.7)	25.0 (16.7)	65.6 (12.5)
	Twice a week	75.0 (25.0)	46.4 (25.0)	70.8 (15.6)	25.0 (25.0)	70.3 (12.5)
	Thrice a week	75.0 (12.5)	60.7 (32.1)	75.0 (12.5)	25.0 (25.0)	65.6 (21.9)
	Four times a week	75.0 (25.0)	53.6 (25.0)	75.0 (13.5)	33.3 (27.1)	62.5 (18.8)
	Kruskal–Wallis H	1.03	8.06	3.82	0.68	4.64
	p	0.794	0.045	0.282	0.879	0.201

*WHOQOL-BREF* World Health Organization Quality of Life-Brief Version, *QoL* Quality of Life, *CKD* Chronic Kidney Disease, *HD* Hemodiaysis

Overall QoL was negatively correlated with PHQ-9 Total (*r*_*s*_ = -0.409, *p* < 0.01), HADS-Anxiety (*r*_*s*_ = -0.314, *p* < 0.01), and HADS-Depression (*r*_*s*_ = -0.432, *p* < 0.01). The physical health dimension was negatively correlated with PHQ-9 Total (*r*_*s*_ = -0.647, *p* < 0.01), HADS-Anxiety (*r*_*s*_ = -0.396, *p* < 0.01), and HADS-Depression (*r*_*s*_ = -0.612, *p* < 0.01). The psychological dimension was negatively correlated with PHQ-9 total (*r*_*s*_ = -0.566, *p* < 0.01), HADS-Anxiety (*r*_*s*_ = -0.477, *p* < 0.01), and HADS-Depression (*r*_*s*_ = -0.694, *p* < 0.01). The social relationships dimension was negatively correlated with PHQ-9 total (*r*_*s*_ = -0.239, *p* < 0.01), HADS-Anxiety (*r*_*s*_ = -0.242, *p* < 0.01), and HADS-Depression (*r*_*s*_ = -0.291, *p* < 0.01). The results were displayed in Table 3.

**Table 3:** Correlation results of the WHOQOL-BREF domains, demographics,and psychometric scales

	1	2	3	4	5	6	7	8	9	10	11
Overall WHOQOL-BREF	1										
PhysicalHealth	0.371**	1									
Psychological	0.490**	0.588**	1								
SocialRelationships	0.239**	0.464**	0.311**	1							
Environment	0.339**	0.224**	0.506**	0.209**	1						
PHQ-9 Total	-0.409**	-0.647**	-0.566**	-0.339**	-0.131	1					
HADS-Anxiety	-0.314**	-0.396**	-0.477**	-0.242**	-0.183**	0.516**	1				
HADS-Depression	-0.432**	-0.612**	-0.694**	-0.291**	-0.276**	0.716**	0.606**	1			
FamilyIncome	0.193**	0.001	0.065	0.100	0.160*	0.099	0.071	0.085	1		
Duration of CKD	-0.038	-0.197**	-0.205**	-0.123	-0.060	0.221**	0.143*	0.250**	-0.027	1	
Duration of HD	0.030	-0.171*	-0.137	-0.122	-0.014	0.168*	0.099	0.192**	-0.53	0.834**	1

*WHOQOL-BREF* World Health Organization Quality of Life-Brief Version, *PHQ9* Patient Health Questionnaire Version 9, *HADS-Anxiety* Hospital Anxiety and Depression Scale-Anxiety Dimension, *HADS-Depression*, Hospital Anxiety and Depression Scale-Depression Dimension, *CKD* Chronic Kidney Disease, *HD* Hemodialysis

1 = Overall WHOQOL-BREF

2 = Physical health

3 = Psychological

4 = Social relationships

5 = Environment

6 = PHQ-9 Total

7 = HADS-Anxiety

8 = HADS-Depression

9 = Family Income

10 = Duration of CKD

11 = Duration of HD

^*^p < 0.05

^**^p < 0.01

30.6% of the variability in overall QoL scores and a 25% increase in predictive capacity were accounted for by the inclusion of PHQ-9 total, HADS-Anxiety, and HADS-Depression scores [F(3, 191) = 22.924, *p* < 0.001]. 48% of the variability in physical health scores and a 36.7% increase in predictive capacity were accounted for by the inclusion of PHQ-9, HADS-Anxiety, and HADS-Depression scores [F(3, 191) = 44.862, *p* < 0.001]. 54.2% of the variability in psychological scores and a 46.7% increase in predictive capacity were accounted for by the inclusion of PHQ-9 total, HADS-Anxiety, and HADS-Depression scores [F(3, 191) = 64.896, *p* < 0.001]. 25.9% of the variability in social relationships scores and a 6.7% increase in predictive capacity were accounted for by the inclusion of PHQ-9, HADS-Anxiety, and HADS-Depression scores [F(3, 191) = 5.745, *p* < 0.001]. 22% of the variability in environmental scores and a 13.4% increase in predictive capacity were accounted for by the inclusion of the PHQ-9, HADS-Anxiety, and HADS-Depression scores [F(3, 191) = 10.906, *p* < 0.001].

The beta coefficients reveal significant associations in our study: higher family income correlates with increased overall QoL, while elevated depression and anxiety scores are linked to decreased QoL across various dimensions. Older age is associated with lower physical health and social relationship scores. Male gender, higher family income, and younger age are linked to better social relationships. Family income positively affects psychological and environmental scores. Higher depression scores adversely affect physical health, psychological well-being, social relationships, and environmental scores. Higher anxiety scores adversely affect psychological aspects of QoL. These findings highlight the intricate interplay between socioeconomic factors, mental health, and various aspects of QoL, underscoring the need for tailored interventions and support systems. The results were displayed in Table 4.

**Table 4:** The hierarchical regression analyses

Model	**Independent variables**	**B**	**T**	**p**	***F***	***df***	***R***^***2***^	***Model p***
1	Family Income	0.234	3.829	0.000	22.924	3, 191	0.306	< 0.001
	PHQ-9 Total score	-0.202	-2.403	0.017				
	HADS-Depression	-0.278	-3.310	0.001				
2	Age	-0.228	-4.234	0.000	44.862	3, 191	0.480	< 0.001
	PHQ-9 Total score	-0.377	-5.164	0.000				
	HADS-Depression	-0.336	-4.616	0.000				
3	Family Income	0.152	3.054	0.003	64.896	3, 191	0.542	< 0.001
	HADS-Anxiety	-0.261	-4.073	0.000				
	HADS-Depression	-0.509	-7.456	0.000				
4	Age	-0.311	-4.851	0.000	5.745	3, 191	0.259	< 0.001
	Gender	0.211	3.333	0.001				
	Family Income	0.192	3.047	0.003				
	HADS-Depression	-0.254	-2.924	0.004				
5	Age	-0.161	-2.441	0.016	10.906	3, 191	0.188	< 0.001
	Family Income	0.218	3.375	0.001				
	HADS-Depression	-0.368	-4.131	0.000				

*PHQ9* Patient Health Questionnaire Version 9, *HADS-Anxiety* Hospital Anxiety and Depression Scale—Anxiety Dimension, *HADS-Depression* Hospital Anxiety and Depression Scale—Depression Dimension

Model 1: Dependent variable Overall Quality of Life/ General Health Score

Model 2: Dependent variable Physical Health Score

Model 3: Dependent variable PsychologicalScore

Model 4: Dependent variable Social Relationships Score

Model 5: Dependent variable EnvironmentScore

## Discussion

In this study, we present novel findings on depression and anxiety prevalence among kidney failure patients undergoing HD in Somalia. Using HADS-Depression, 37.5% reported mild to moderate depression, compared to 13.5% with PHQ-9. Anxiety symptoms were reported by 7% of participants. Depression and anxiety scores were significantly correlated with QoL dimensions. Age and depression predicted physical health, family income, depression, and anxiety predicted psychological well-being, age, gender, family income, and depression predicted social relationships, and age, family income, and depression predicted environmental well-being.

In our study, we observed variations in the point prevalence of depression and anxiety among kidney failure patients undergoing HD when compared to different study populations. The depression prevalence rate in our study aligns with findings from various African studies, reporting rates ranging from 34.5% to as high as 72% [8, 9, 24]. The 37.5% prevalence of depression in this study is also consistent with a meta-analysis that estimated a 39.3% prevalence rate [25]. However, it is important to note that the 7% prevalence of anxiety in our study was notably lower than the estimated prevalence of 38% (with a range of 12% to 52%) reported in a comprehensive systematic review [26].

The high depression rates among kidney failure patients undergoing HD in our study can be linked to economic struggles, including job loss and low family income. We found a significant correlation between low family income and increased depression levels. Many patients could only afford a fraction of their HD sessions due to financial constraints, adding to their burden. This highlights the urgent need for healthcare systems in Somalia to address financial barriers and improve access to quality treatment. Targeted interventions are crucial to alleviate the psychological distress faced by these patients and enhance their overall well-being.

Our study emphasizes the critical impact of socioeconomic factors, notably family income and socioeconomic status, on depression among HD patients, aligning with previous research findings [8, 27–30]. Addressing these economic challenges is vital, as financial stress profoundly affects HD patients' mental well-being and QoL. Additionally, our study sheds light on the overlooked issue of anxiety, revealing its significant independent contribution to reduced QoL among HD patients, beyond its connection with depression [31, 32]. The observed variations in anxiety and depression prevalence among Somali HD patients might stem from cultural factors and the unique resilience of this population, emphasizing the need for culturally sensitive interventions [31]. Previous research has indicated that anxiety can detrimentally affect the QoL of HD patients independently, rather than solely as an overlapping symptom of depression [32]. Our study extends this understanding, revealing that anxiety serves as a significant contributor to diminished QoL among HD patients. These insights underscore the urgency of tailored mental health support programs for HD patients in similar settings, acknowledging both depression and anxiety as distinct concerns within this context.

Our study reveals the significant impact of depression and anxiety on HD patients' QoL, particularly in social relationships and physical health dimensions. These findings underscore the necessity for targeted interventions to enhance the well-being of HD patients. Interestingly, our study's prevalence rates of low QoL were lower than those reported in other African HD populations [8, 33–37], possibly due to cultural and psychological resilience factors within the Somali population or differences in perception and reporting. Further research is essential to explore these disparities and delve into the cultural and psychological factors influencing QoL experiences in Somali HD patients. Such insights are crucial for developing culturally sensitive interventions and support systems tailored to the specific needs of this population. Additionally, investigating the interplay between mental health, cultural factors, and QoL in HD patients can provide valuable knowledge for healthcare providers and policymakers in similar contexts.

Our study on HD patients highlighted significant negative correlations between depression and anxiety scores and various aspects of QoL, emphasizing the pivotal role of addressing mental health issues in enhancing overall well-being. Depression scores (PHQ-9) and anxiety scores (HADS-Anxiety) were closely linked, emphasizing the need for comprehensive strategies to manage both conditions. Socioeconomic factors and treatment duration were found to be crucial determinants of patients' well-being. Lower family income and longer durations of renal failure and HD treatment were associated with lower QoL, diminished physical health, and poorer psychological well-being, accompanied by higher depression and anxiety levels. Prolonged HD treatment correlated with lower physical well-being and higher depression scores, indicating its adverse impact. These findings align with previous research [8, 28, 38, 39], highlighting the risk of heightened depression and anxiety in CKD patients undergoing extended HD. Higher income levels positively influenced QoL, consistent with previous research [30], emphasizing the importance of addressing financial stress. In summary, our study illuminates the intricate relationship between socioeconomic factors, treatment duration, and the mental and physical health of CKD patients undergoing HD, emphasizing the need for tailored interventions to enhance the overall well-being and QoL of this vulnerable patient group.

Our study shed light on the significant impact of socioeconomic factors and mental health on the QoL of HD patients, especially those with low family income. Low income emerged as a strong predictor of lower QoL, affecting overall well-being, psychological, social, and environmental dimensions. This emphasizes the financial challenges faced by our patient cohort, many of whom rely on hospital support for HD sessions. Depression, measured by PHQ-9 and HADS-D, proved influential in predicting QoL and physical health, highlighting its pervasive impact. Anxiety and depression, assessed by HADS-Anxiety and HADS-Depression, significantly affected psychological well-being. Depression also impacted social relationships and environmental well-being, irrespective of demographics. Our findings underscore the need for proactive screening, early intervention, and comprehensive care to address depression and anxiety in HD patients. By managing these emotional challenges, healthcare providers can substantially enhance QoL and life satisfaction among HD patients, emphasizing the importance of holistic patient support.

This study, focused on anxiety and depression's impact on the QoL of Somali HD patients, has limitations. We couldn't assess factors like nutritional status, electrolytes, BMI, and inflammation markers due to financial constraints. The small sample size limits generalizability, and the cross-sectional design prevents establishing causality or tracking changes. Future research should employ larger samples, longitudinal approaches, and comprehensive data collection to overcome these limitations and enhance understanding.

In summary, our study emphasizes the crucial impact of depression, anxiety, and family income on the QoL of Somali HD patients. Routine emotional screening and effective management are vital for enhancing patient well-being. Larger studies are needed for detailed analysis, and a multidisciplinary healthcare approach is recommended for comprehensive patient care .

## Data Availability

The datasets used and/or analysed during the current study are available from the corresponding author on reasonable request.
